# Dual-database Bibliometric Analysis Combined with Gephi-based Network Visualization of Artificial Intelligence Applications in the Identification and Diagnosis of Thyroid Space-occupying Lesions

**DOI:** 10.2174/0115734056466850260610101215

**Published:** 2026-06-16

**Authors:** Shilin Wen, Weiqing Qian, Yuyang Su, Xiaohan Cao, Toutou Chen, Weidong Gong, Xiaocan Lei

**Affiliations:** 1 Clinical Anatomy and Reproductive Medicine Application Institute, The First Affiliated Hospital, Hengyang Medical School, University of South China, Hengyang 421001, China; 2 Affiliated Hengyang Hospital of Hunan Normal University & Hengyang Central Hospital, Hengyang, 421001, Hunan, China

**Keywords:** Thyroid space-occupying lesions, Artificial intelligence, Hotspot, Bibliometrics, Multimodal analysis, Precision medicine, H index

## Abstract

**Introduction/Objective:**

Accurate differentiation between benign and malignant thyroid space-occupying lesions is critical for clinical decision-making and early treatment planning. Existing diagnostic methods, including ultrasound and fine-needle aspiration biopsy, are constrained by observer dependence and procedural invasiveness. Recent advances in Artificial Intelligence (AI) have provided objective and non-invasive alternatives for thyroid lesion assessment. This study aimed to delineate the research landscape, identify major hotspots, and summarize recent progress in the application of AI to thyroid space-occupying lesions.

**Methods:**

Publications issued between 2006 and 2025 were retrieved from WOSCC and PubMed, yielding 1546 records for screening. Bibliometric analyses were conducted using CiteSpace and VOSviewer, and network visualization was performed using Gephi. Country and institutional contributions, author collaborations, journal distribution, keyword co-occurrence, and knowledge unit co-occurrence networks were systematically examined.

**Results:**

Publication output increased sharply, with 86% of the included studies published within the past 5 years. China (371 articles, 56.9%) and the United States (109 articles, 16.7%) were the leading contributors and central nodes in the collaboration networks. Shanghai Jiao Tong University, the Chinese Academy of Sciences, and Sun Yat-sen University were the main contributing institutions, and their research efforts mainly involved clinical problems, algorithm development, and global multicenter validation, respectively. Major hotspots included deep learning-based thyroid nodule detection and classification, multimodal feature fusion, model optimization, and clinical risk stratification. Keyword analysis identified deep learning, classification, and risk stratification as core themes.

**Discussion:**

AI research in thyroid diagnosis is progressing from technical exploration to clinically oriented applications. China led the publication output, whereas the United States showed stronger international collaboration. The field has also shifted from simple classification to integrated risk stratification and multimodal analysis, reflecting the increasing alignment with precision medicine.

**Conclusion:**

This study outlines the bibliometric profile of AI applications in thyroid space-occupying lesion identification and identifies gaps in collaboration and topic diversity. Broader cross-national cooperation may enhance research quality and support future research and clinical practice.

## 1 INTRODUCTION

Thyroid disease is one of the most common endocrine disorders worldwide and has long been a major concern in clinical practice [1]. Among thyroid disorders, thyroid space-occupying lesions are frequently encountered and are generally classified as benign or malignant according to their pathological nature. Benign lesions (*e.g*., nodular goiter and thyroid adenoma) usually progress slowly and are associated with favorable prognoses [2]. Malignant lesions, mainly thyroid cancer, exhibit heterogeneous biological behavior. Although most differentiated thyroid cancers, including papillary thyroid carcinoma, have a relatively favorable prognosis [3, 4], some subtypes remain highly invasive and carry a high risk of recurrence, thereby seriously affecting patients’ quality of life [5]. Therefore, the accurate differentiation between benign and malignant thyroid space-occupying lesions is not only the first step in clinical decision-making but also a prerequisite for early intervention and precise tumor management. The detection rate of thyroid nodules in the general population can reach 50–60% [6, 7], and the global incidence of thyroid cancer as the most common endocrine malignancy has continued to increase over recent decades [8-10] and thyroid cancer, as the most common type of endocrine malignancy, has seen a continuous increase in global incidence over the past few decades [11]. Among the pathological subtypes of thyroid cancer, Papillary Thyroid Cancer (PTC) is predominant [12-14]. Its early and accurate detection and classification are critical for treatment planning, prognostic assessment, and preservation of patients’ quality of life [15,16]. Currently, the clinical diagnosis of thyroid nodules mainly relies on high-resolution ultrasound imaging and Fine-Needle Aspiration Biopsy (FNAB) [17-19]. However, ultrasound interpretation is strongly influenced by physician experience and remains subjective. Moreover, FNAB is invasive, and approximately 10–30% of cases cannot be clearly classified as benign or malignant [20, 21], complicating clinical decision-making. Thus, accurate differentiation of benign and malignant thyroid space-occupying lesions is critical for timely intervention, precise management, and improvement of patient prognosis and quality of life [15, 16].

With the rapid development of Artificial Intelligence (AI), Machine Learning (ML), Deep Learning (DL) [22, 23], and Transfer Learning (TL) [24, 25] have shown strong performance in medical image recognition and classification. Through Convolutional Neural Networks (CNNs) and related architectures [26, 27], these methods can automatically extract multidimensional ultrasound features, including nodule morphology, echo characteristics, and calcification patterns, thereby enabling end-to-end benign/malignant discrimination, reducing human bias, and improving diagnostic consistency. Previous studies have reported that AI-based thyroid nodule detection systems can achieve or even exceed the diagnostic performance of experienced radiologists across multiple independent datasets [28, 29]. In addition, TL can alleviate the limitation of small medical imaging datasets by using models pre-trained on large-scale natural image datasets, such as ImageNet [30, 31], thereby improving model convergence and generalization in thyroid space-occupying lesion diagnosis. These technological advances have promoted the practical implementation of Computer-Aided Diagnosis (CAD) systems and provided new approaches for personalized medicine and early screening.

Although substantial progress has been made in AI-based detection and classification of thyroid mass lesions [32], this field remains in a stage of rapid development. Related research topics are still dispersed, technical pathways are diverse, and systematic evidence on the overall knowledge structure, collaboration networks, and developmental trends remains limited. As an interdisciplinary approach integrating mathematics, statistics, and information science, bibliometrics can identify research hotspots, evolutionary trajectories, and frontier directions through quantitative analysis and visual representation of large-scale academic literature [33, 34]. Although several reviews have examined AI applications in general medical imaging [35-37], a comprehensive and systematic overview of the knowledge structure, collaboration networks, and evolving research frontiers specific to AI-assisted diagnosis of thyroid space-occupying lesions is still lacking. Additionally, the exponential growth of literature requires more systematic methods for reviewing and analyzing research trends within this domain [38]. This gap limits researchers’ ability to understand the overall landscape of the field and identify its future directions. Therefore, this study conducted a comprehensive bibliometric analysis of global research on the use of AI in the diagnosis of thyroid space-occupying lesions.

### 1.1 Key Research Contributions

Key research contributions of the study are as follows:

- Systematic and quantitative mapping of the knowledge landscape in this emerging field, with key research hotspots and evolutionary trends identified.
- Multilevel analysis of global collaboration networks across countries, institutions, and authors, with structural patterns of scientific cooperation and potential gaps clarified.
- Identification of the foundational knowledge base and emerging frontiers, providing empirical evidence for future research directions and strategic resource allocation.

The remainder of this paper is organized as follows: Section 2 describes the materials and methods, including the dual-platform search strategy, data collection procedure, and bibliometric tools used for analysis. Section 3 reports the bibliometric results, covering publication trends, country, institutional, and author contributions, and research hotspots and frontiers. Section 4 discusses the findings within the broader research landscape. Section 5 outlines the strengths and limitations of this study. Section 6 summarizes the key findings and proposes future research directions.

## 2 MATERIALS AND METHODS

### 2.1 Data Sources and Retrieval Strategy

A dual-platform retrieval strategy was adopted to ensure both the comprehensiveness and accuracy of the literature search. The Web of Science Core Collection (WoSCC) was selected as the primary data source for bibliometric analysis because of its established indexing of high-quality academic literature. This selection was justified by three main reasons. First, the strict journal-selection criteria of WoSCC based on Bradford’s Law and Garfield’s Law ensured the academic quality of the included publications. This could be particularly relevant to the interdisciplinary and frontier-oriented field of artificial intelligence in the diagnosis of thyroid space-occupying lesions. Second, WoSCC provides standardized bibliographic and citation records, including information on authors, affiliations, and references. These structured data align well with the analytical requirements of bibliometric tools such as CiteSpace and VOSviewer and can be exported directly for analysis, thereby reducing the potential information bias caused by data-format conversion. Third, the broad disciplinary coverage of WoSCC allows studies from computer science, engineering, medicine, imaging, and other related fields to be comprehensively captured [39-41]. Furthermore, its rigorous and standardized literature selection process enhances the scientific validity and credibility of the research data [42, 43]. Because clinical trial-related information is only partly covered by the primary Web of Science database, PubMed was also searched to supplement clinical research data and assess the progress of clinical studies in this field. The detailed search strategies for both databases are provided in **Supplementary Material 1**.

Based on this, a retrieval strategy was designed to cover the core concepts of AI and its typical application scenarios, including lesion detection, tissue classification, and clinical diagnosis. Target thyroid diseases were also included in the search framework, comprising thyroid nodules, thyroid carcinoma, and thyroid space-occupying lesions.

### 2.2 Data Collection and Analysis

**Fig. (1):**
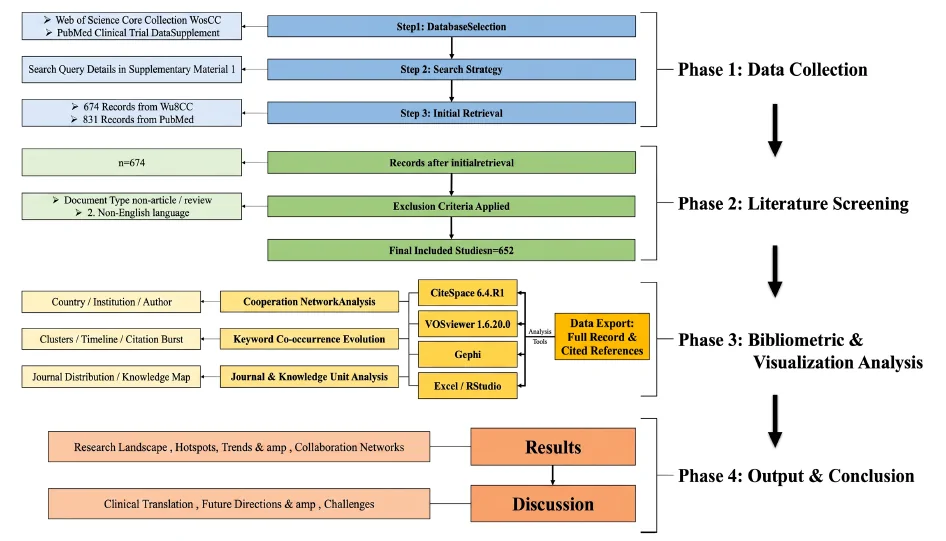
Flow chart of the experiment.

The initial search retrieved 674 literature records. All records were exported from WoSCC in plain-text format as full records, including cited references, for subsequent analysis. To ensure a rigorous screening process, the following criteria were applied (Fig. **1**). The inclusion criteria were publications issued between January 1, 2006, and October 13, 2025; original English research articles and review articles; and a research focus on artificial intelligence in the diagnosis of thyroid space-occupying lesions. The exclusion criteria involved incomplete information or unavailable full text, duplicate records, publications outside the predefined document categories, and studies whose core content was unrelated to artificial intelligence in the diagnosis of thyroid space-occupying lesions. Literature screening was performed independently by two researchers. After the initial retrieval of 674 records, 19 records (2.8%) were excluded due to document type (editorials, letters, and conference abstracts), and 3 records (0.46%) were excluded because they were non-English publications. After screening, 652 papers were included in the final analysis. Disagreements were resolved through discussions with a third expert. The complete screening workflow is presented in Fig. (**1**), which details the number of records identified, screened, and excluded at each stage, thereby ensuring methodological transparency and reproducibility.

To systematically characterize the research landscape, collaboration networks, knowledge base, research hotspots, and frontier evolution in AI-assisted diagnosis of thyroid space-occupying lesions, a multilevel analytical framework was adopted from both macro- and micro-level perspectives.

CiteSpace (version 6.4.R1) and VOSviewer (version 1.6.20.0) were selected as the primary bibliometric visualization tools. These tools were adopted because of their complementary analytical functions. VOSviewer is well-suited for constructing and visualizing co-authorship networks involving countries, institutions, and authors, as well as keyword co-occurrence networks. It can provide intuitive distance-based clustering and high-quality graphical output [38]. CiteSpace is particularly suitable for detecting research frontiers and evolutionary trends through citation burst detection and timeline visualization, allowing emerging topics and paradigm shifts to be identified [44]. The integration of these tools enabled a comprehensive multidimensional analysis of the knowledge structure, collaboration patterns, and developmental trajectories of AI-assisted thyroid lesion diagnosis.

Within the Java Runtime Environment, VOSviewer and CiteSpace were used to construct and interpret visual maps of core networks, including countries, institutions, authors, keyword co-occurrence, and paper co-citation networks. Excel was used to organize basic data and perform descriptive statistics, whereas RStudio and related scientometric packages were used for supplementary calculations, including the H-index, and for the generation of additional charts.

The overall workflow of this study, from data sources to final results, is illustrated in **Supplementary Material 2**.

## 3 RESULTS

### 3.1 Analysis of the Publication Volume Trends

**Fig. (2):**
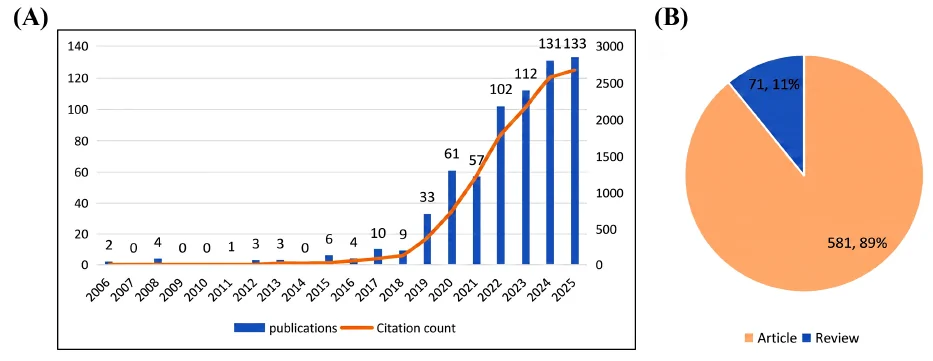
Annual trends in publications and citations on AI for thyroid disease diagnosis (2006-2025). This figure includes (**A**) Temporal distribution of annual publication volume and citation frequency, and (**B**) Proportional distribution of literature types (article *vs*. review).

Annual publication output and citation counts are key quantitative indicators for evaluating the developmental dynamics, academic influence, and maturity of a research field. Using WoSCC data from 2006 to 2025, this study examined the evolutionary trajectory of artificial intelligence in the diagnosis of thyroid space-occupying lesions (Fig. **2A**).

The analysis indicated the transition of this field through three successive stages over the past two decades: dormancy, initial development, and accelerated growth. This trajectory is broadly consistent with the technology maturity curve. The first stage (2006–2018) was mainly characterized by technological and conceptual validation. During this period, the annual publication output remained below 10 papers and even reached zero in some years, suggesting that research activity was still fragmented and that a stable academic community had not yet formed. The major breakthrough reported by DAW [45] may have contributed to the rise in annual citations from 54 in 2016 to 125 in 2018, indicating that early technological progress began to draw academic attention to this field. The second stage (2019–2021) reflected field formation and rapid expansion. The annual publication output increased from 33 papers in 2019 to 102 papers in 2021, representing a growth of more than 200% within three years. Annual citations also increased substantially, reaching 1246 in 2021. These changes suggest that the field has moved from an emerging innovation phase to an early growth phase, accompanied by a clearer research framework and more active scholarly discussion. The third stage (2022–2025) was marked by rapid expansion and increasing mainstream recognition. The annual publication output remained at a high level, and the incomplete statistics for 2025 had already reached 133 papers. More importantly, annual citations grew faster than publication output, exceeding 2000 in 2023 (2179 citations). This pattern suggests that research findings in this field have gained broader academic recognition and application, with their influence continuing to expand within the field of medical artificial intelligence.

Regarding literature type distribution, the 652 included papers comprised 581 research articles and 71 reviews, accounting for 89% and 11% of the total, respectively (Fig. **2B**). The dominance of original research articles indicates that the field remains in an active phase of knowledge production, technological validation, and methodological updating. Additionally, the gradual emergence of review articles suggests that the rapidly expanding evidence base is beginning to be summarized and integrated to guide future research directions.

### 3.2 National Perspectives on Artificial Intelligence in Thyroid Disease Diagnosis Research

The 652 papers included in this study represented research outputs from multiple countries worldwide. The visualization of national differences, institutional distributions, author productivity, and collaboration patterns provides an overview of the relative influence and cooperative network characteristics of countries, institutions, and researchers. It also offers a structural basis for assessing the development process and resource allocation in this field.

The publication output, citation counts, and cooperation intensity at the country level are presented in Table **1**. Based on publication output, citation count, H-index, average citations, and total link strength, the leading countries in AI-assisted thyroid diagnosis were China, the United States (US), South Korea, Italy, and India. China ranked first, with 371 papers and 6149 total citations, indicating extensive research coverage and high productivity in this field. The US ranked second in publication output, with 109 papers, and showed strong academic influence, as reflected by 2987 total citations and an average citation count of 27.4 South Korea and Italy also showed relatively high average citation counts of 23.6 and 23.77, respectively. India ranked fifth in terms of publication output. However, its average citation count was 8.38, suggesting that its research influence requires further improvement. Regarding international cooperation, the US had the highest total link strength (86), indicating its central position in the global collaboration network.

China had a total link strength of 71, which was lower than that of the United States but still reflected active international collaboration. Overall, AI-assisted thyroid diagnosis has developed into a global research field centered mainly in China and the US, with additional contributions from South Korea, Italy, and India. Further improvements are possible in both research quality and international cooperation. To characterize the global collaboration structure more comprehensively, multiple complementary visual maps were used to examine the field from the perspectives of macroscopic distribution, core structure, and clustering patterns.

**Fig. (3):**
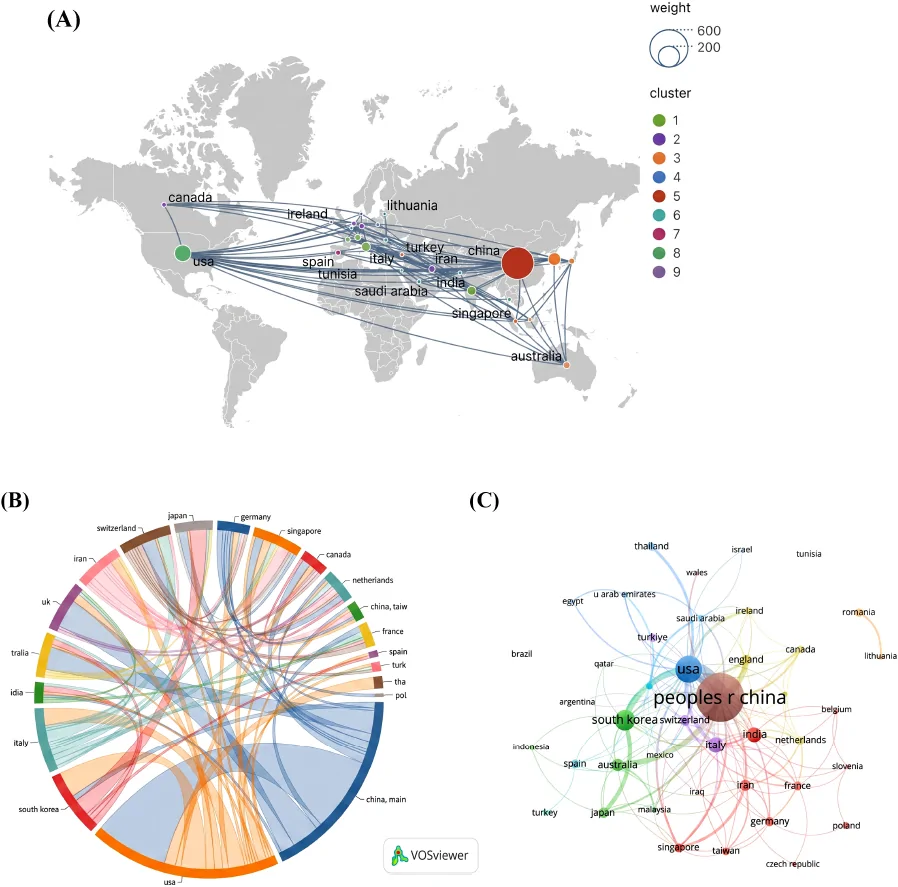
(**A**)World map showing the contributions, (**B**) chord diagram of inter-country co-authorship, and (**C**) Network visualization diagram of inter-country co-authorship of various countries to research in the field of drug target identification.

The national co-occurrence clustering map shows that research in this field has been conducted on a global scale. Different colors denote distinct collaboration clusters (Fig. **3A**). A multicore cooperation structure was observed, including a China-centered cluster linked with Asia-Pacific countries or regions such as Singapore and South Korea, and a US-centered cluster connected with Canada, Ireland, and several European countries. This pattern indicates a multipolar global collaboration network rather than a single-center structure.

The national cooperation network graph further quantifies the cooperation intensity among major contributing countries through the thickness of the connecting chords (Fig. **3B**). The length of each circular arc corresponds to the total publication output of a country, with China and the US showing the most prominent arcs. The thickness of each chord represents the intensity of international cooperation. The US presented strong links with several countries, including Canada, the United Kingdom (UK), and the Netherlands, reflecting frequent and extensive international collaboration. In contrast, although China also had international cooperation links, the breadth and intensity of these links remained relatively limited. Therefore, future strategies should focus on deepening and expanding China’s international collaboration network.

In the national cooperation network diagram, the node size represents the national publication output (Fig. **3C**). China and the US appeared as the largest nodes, confirming their status as core countries in this field. The connections between nodes indicate intercountry collaboration, whereas the link thickness represents cooperation intensity. The diagram depicts a collaboration topology centered on China and the US, with additional links involving Canada, Australia, Singapore, Italy, and other countries.

### 3.3 Institutional Landscape of Artificial Intelligence Applied in the Field of Thyroid Disease Diagnosis

**Fig. (4):**
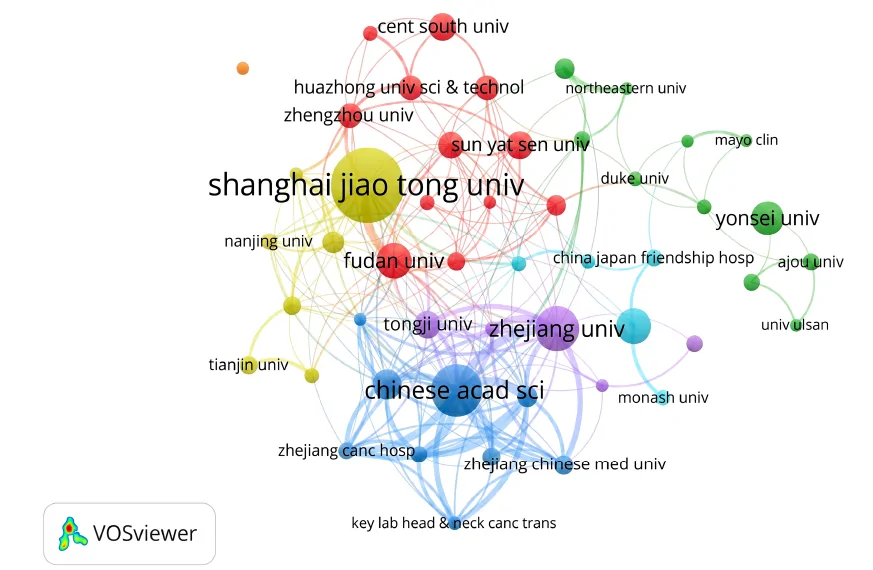
Network visualization map of inter-institutional co-authorship, colored by clustering.

Our literature analysis revealed a diverse pattern of institutional collaboration in this field. The institutional cooperation network was constructed using VOSviewer, through which 1201 institutions were identified (Fig. **4**). When the publication threshold was set to 7, 49 institutions met the inclusion criteria. The top 10 institutions by publication output were mainly located in China, with Yonsei University in South Korea also included, forming a dense Asia-Pacific-centered research collaboration network (Table **2**). Shanghai Jiao Tong University ranked first with 40 publications, indicating strong institutional productivity. The Chinese Academy of Sciences ranked second with 37 publications and had the highest total link strength of 85, highlighting its central role as a collaboration hub and its strong capacity for resource integration and international cooperation. In terms of research quality and impact, Sun Yat-sen University showed considerable academic influence, with a mean citation rate of 37.24 and an H-index of 13. This suggests that its research outputs have been widely recognized by peers.

The global institutional collaboration structure is further presented in the institutional cooperation network map (Fig. **4**). In this map, institutions are represented as nodes, the node size corresponds to publication output, and line thickness reflects the intensity of inter-institutional cooperation. Cluster analysis of the network identified three core research clusters in AI-assisted diagnosis of thyroid space-occupying lesions, each with distinct regional features and research orientations. Cluster 1, the core clinical research network in China, was centered on leading Chinese medical universities and affiliated hospitals, including Shanghai Jiao Tong University, Zhejiang University, and Fudan University. Supported by abundant clinical resources, this cluster focused on the application of AI to clinical problems, such as ultrasound imaging diagnosis, benign/malignant differentiation, and risk prediction of thyroid nodules, especially PTC. Thus, it represents a major force driving clinical translation and application validation of AI technology. Cluster 2, the interdisciplinary basic research and algorithm innovation cluster, was centered on the Chinese Academy of Sciences, which showed the highest link strength and broad connections with universities and hospitals in China and abroad. This cluster focused on frontier methodological research, including AI algorithm development, model construction, and cross-omics data fusion, thereby providing original methodological support for the field. Cluster 3, the high-level medical center collaboration cluster between China and South Korea, was centered on internationally recognized medical centers, including Yonsei University in South Korea and Zhejiang Cancer Hospital in China. This cluster mainly involved multicenter, high-quality clinical cohort studies aimed at developing and validating AI diagnostic models with strong generalizability. Its characteristics included intensive collaboration, shared research-standard development, and the promotion of international recognition of research outputs.

Together, these three core clusters reflected a functional division of labor among clinical problem orientation, basic algorithm innovation, and international multicenter validation. This structure could provide a solid foundation for advancing the field from theoretical method development to clinical application. These clusters represented not only a geographical or institutional distribution but also a functional organization of the research community. Cluster 1, dominated by leading Chinese medical universities, used large-scale clinical data to identify and update key clinical problems, thereby maintaining the clinical relevance of research. Cluster 2, led by the Chinese Academy of Sciences, provided the methodological momentum through algorithmic advances designed to improve diagnostic accuracy and efficiency. Cluster 3, represented by collaborations between Yonsei University and Chinese cancer hospitals, served as a critical validation layer by testing the generalizability and robustness of AI models across multicenter settings. Through collaboration links and resource exchange among these clusters, the field could advance from algorithm development to clinical validation and, ultimately, to wider clinical adoption.

### 3.4 Author Productivity and Collaboration Patterns

**Fig. (5):**
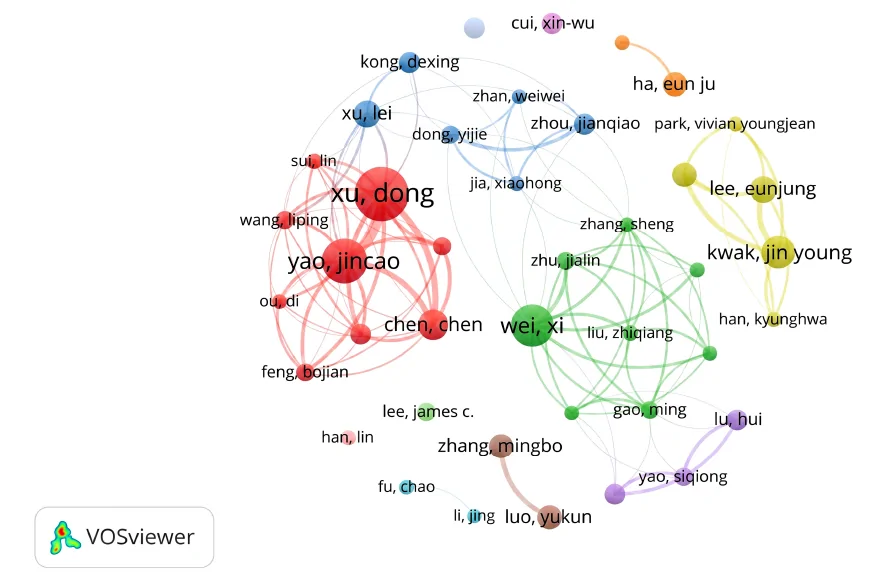
Visualization of author collaboration network. Network visualization diagram of author co-authorship, colored by clustering.

Author analysis is a conventional dimension for constructing knowledge maps in scientific research and provides an important approach for identifying representative scholar groups and evaluating their core research strengths in a specific field. In the research system of AI-assisted diagnosis of thyroid space-occupying lesions, high-productivity authors showed a clear pattern in publication output and academic influence (Table **3**). Xu, Dong from the Chinese Academy of Sciences, ranked the first with 18 publications, followed by Yao, Jincao from the Chinese Academy of Sciences and Wei, Xi from Tianjin Medical University, with 16 and 14 publications, respectively. These authors formed a core output group in this field. In terms of academic influence, Wei, Xi showed strong performance, with an H-index of 11 and 280 total citations. Xu, Dong had the highest total citation count, with 317 citations, but his H-index was slightly lower than that of Wei, Xi, at 10, indicating that the sustained influence of his research outputs may still have room for further development. The collaboration structure and cluster characteristics of authors were further examined using an author collaboration network visualization (Fig. **5**). In this graph, authors are represented as nodes; node size indicates publication output, line thickness reflects cooperation intensity, and color clustering distinguishes different collaboration groups. The network cluster analysis indicated that several distinct and cohesive author collaboration clusters had formed, reflecting clear group-based cooperation and a structured division of research roles Fig. (**5**). Cluster 1, the core research team in China, was centered on Xu, Dong, Yao, Jincao, and Chen, all from the Chinese Academy of Sciences. This group formed a close collaboration network, supported by national-level research platforms, focused on advanced AI algorithm development and multimodal feature fusion, making it an important driver of technological innovation in this field. Cluster 2, the high-level clinical research collaboration group between China and South Korea, was represented by Kwak, Jin Young, Lee, Eunjung, and Yoon, Jung Hyun from Yonsei University in South Korea, together with Chinese scholars such as Luo, Yukun from Southern Medical University. This group formed a cross-national academic–industry–research collaboration network and mainly focused on multicenter clinical imaging data to develop and validate AI diagnostic models with strong generalizability. Its international cooperation model showed a clear orientation toward clinical translation. Cluster 3, the cross-continental collaborative team between China and Sweden, was connected through Xu, Lei from the Karolinska Institute in Sweden, and several Chinese scholars. Although this cooperation remained limited in scale, it reflected an emerging pattern of cross-continental knowledge exchange and provided channels for introducing international methodological advances and validation standards.

Although Chinese scholars dominated the total publication output, their international cooperation network remained relatively localized and was mainly concentrated in the Asia-Pacific region. Broader and deeper collaboration with North America, Europe, and other regions may be required. In contrast, Korean scholars, despite their smaller overall output, showed a stronger tendency towards international collaboration. Overall, the author collaboration network analysis not only identified high-productivity scholars and core teams in this field but also revealed a research pattern dominated by regional clusters and an emerging form of cross-national academic–industry–research cooperation. In the future, further expansion and deepening of cross-regional and interdisciplinary collaboration, particularly closer integration between algorithm experts and clinicians, will be essential for advancing this field from methodological innovation to clinical validation.

### 3.5 Keyword Evolution and Research Frontiers

**Fig. (6):**
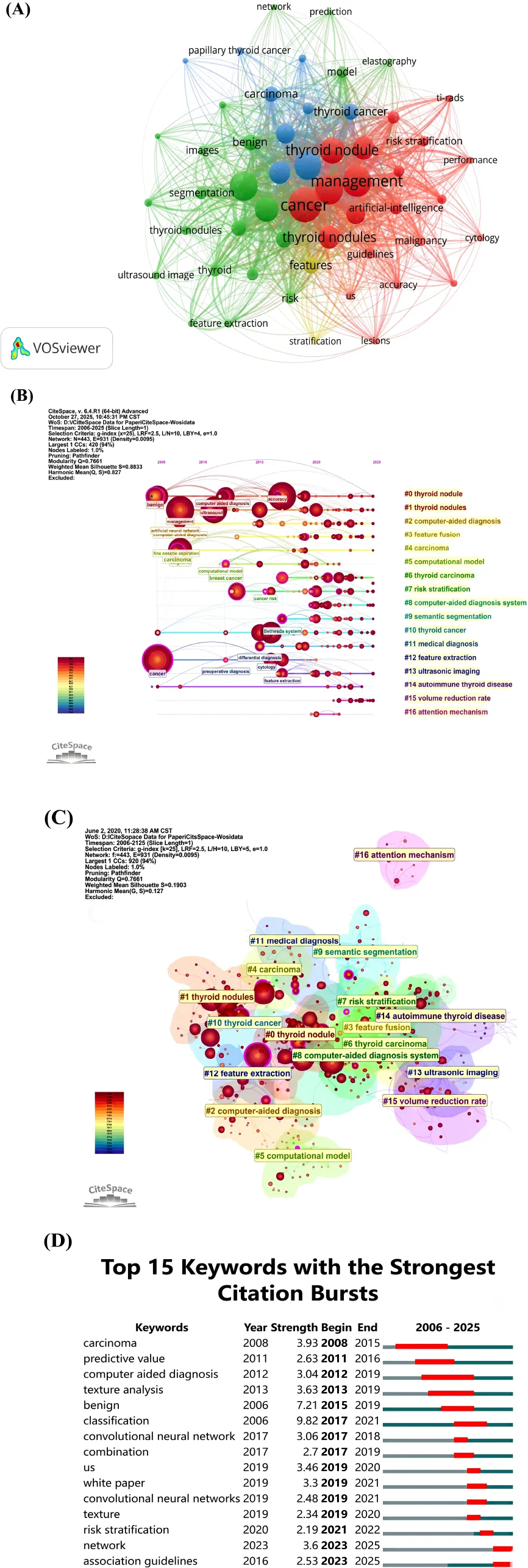
Keyword visualization. This figure includes (**A**) Keyword co-occurrence clustering network diagram, (**B**) Keyword timeline chart, (**C**) CiteSpace keyword clustering map, and (**D**) Keyword citation burst diagram.

Keyword co-occurrence analysis was performed using VOSviewer. A total of 1920 keywords were identified, and 49 keywords met the threshold of 18 occurrences. Based on this, a keyword co-occurrence clustering network was generated (Fig. **6A**). The color and spatial distribution of nodes indicate the relative proximity among keywords. “Artificial intelligence” and “classification” were closely connected with clinically relevant terms, such as “ultrasound image” and “thyroid nodule,” forming the central region of the network. In addition, “risk stratification”, “guidelines, “accuracy”, and “malignancy” formed another closely connected community, reflecting the clinical application direction of risk assessment and management guidance. Table **4** lists the top 10 core keywords according to their frequency and total link strength. Among them, “cancer” (frequency 123, link strength 1168) and “classification” (frequency 117, link strength 679) demonstrated high frequencies and strong link strengths, indicating that the precise classification of thyroid cancer is a central objective in this field. “Artificial intelligence” (frequency 179, link strength 669) served as the technical foundation and, together with “computer-aided diagnosis” (frequency 100, link strength 361), constituted the methodological core of this field. Meanwhile, the high frequency of “accuracy” reflected the continued emphasis on diagnostic performance as a key evaluation criterion. To further clarify topic evolution, a multidimensional analysis was conducted using CiteSpace. The timeline map Fig. (**6B**) illustrates the temporal evolution of research topics. Although core concepts such as “cancer” and “classification” appeared early and persisted over time, AI-related methods have received increasing research attention since approximately 2018, forming an active and continuous research trajectory. The keyword clustering map (Fig. **6C**) classified closely related keywords using the LLR algorithm and generated a clustering network with high modularity (Q = 0.7651) and mean silhouette value (S = 0.8983). The clustering labels, including #0 thyroid nodules, #1 computer-aided diagnosis, #2 feature fusion, #3 carcinoma, #4 computational model, #5 thyroid carcinoma, #8 computer-aided diagnosis system, #9 semantic segmentation, #12 feature extraction, and #16 attention mechanism, systematically outline three core pillars of this research field. First, the field was anchored in clinical problems. Clusters such as #0 thyroid nodules, #3 carcinoma, and #5 thyroid carcinoma directly corresponded to the disease entities addressed in clinical practice. These clusters clarified that the central research goal could meet diagnostic needs related to thyroid space-occupying lesions and indicated that subsequent technological development should ultimately serve clinical tasks, such as benign/malignant differentiation and disease grading. Second, the field was driven by technical tasks. With #1 computer-aided diagnosis as the overall objective, technical nodes such as #9 semantic segmentation and #12 feature extraction formed a complete technical chain from image analysis, represented by lesion segmentation, to feature mining, represented by key information extraction, and then to decision support through auxiliary diagnosis. This chain illustrated how clinical problems were translated into technical tasks and how clinical needs were linked to methodological innovation. Third, methodological models increasingly incorporated frontier strategies, including #2 feature fusion, #4 computational model, and #16 attention mechanism, to address core technical limitations. These strategies can support information integration through multimodal feature fusion, recognition of complex lesions using advanced computational models such as DL, and improved model interpretability using attention mechanisms. The keyword citation burst map (Fig. **6D**) identified keywords with sharp increases in citation frequency during specific periods, thereby revealing emerging trends and research frontiers. The analysis of the strongest burst keywords revealed a clear shift in the research focus. From 2006 to 2016, keywords such as “carcinoma”, “texture analysis”, and “computer-aided diagnosis” dominated, reflecting early exploratory studies based on hand-crafted features and conventional machine learning. From 2017 to 2021, burst keywords such as “convolutional neural network”, “deep learning”, and “classification” indicated the widespread adoption of end-to-end deep learning models, which substantially improved diagnostic performance. Since 2022, emerging keywords such as “risk stratification”, “association guidelines”, and “attention mechanism” have indicated a paradigm shift towards clinical relevance, interpretability, and alignment with established clinical practice guidelines. This evolutionary trajectory reflects technological maturation and highlights the transition of AI from a lesion-detection tool to a clinical decision-support system.

### 3.6 Knowledge Unit co-occurrence Network

**Fig. (7):**
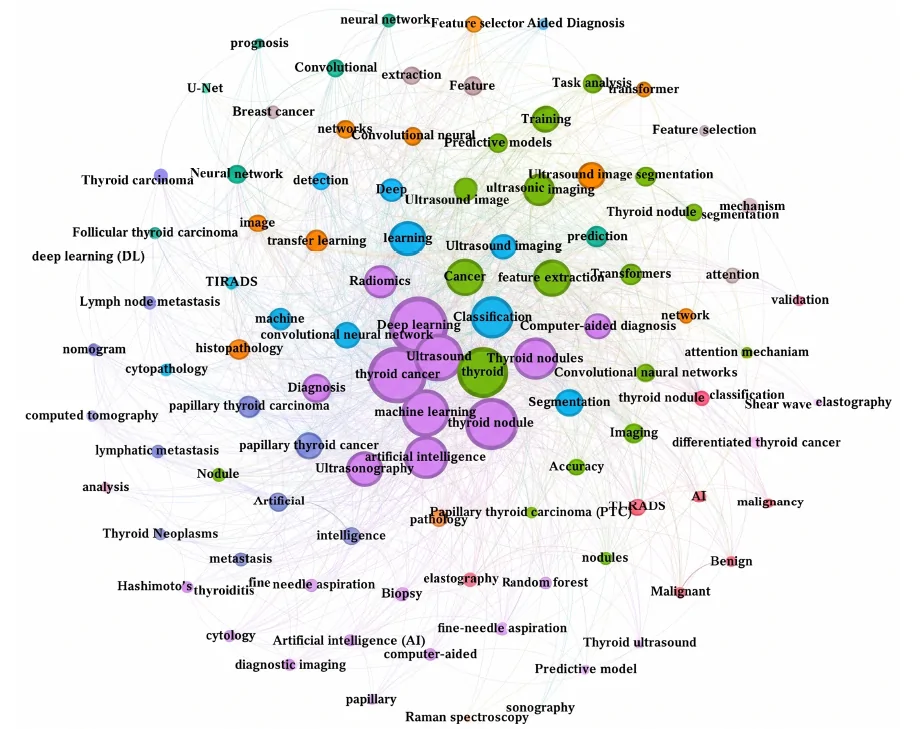
Visualized clustering network of WoSCC keywords.

According to the Gephi layout settings used in this study (region area: 10000.0, gravity constant: 5.0, speed: 1.0), the spatial configuration compressed the distances between nodes while maintaining overall network stability (Fig. **7**). This layout improved the visual separation of cluster boundaries and clarified the hierarchical relationships among clinical problems, technical methodologies, and validation applications. These results support the structural correspondence with the three functional clusters identified in the preceding sections: clinical-driven research, algorithm innovation, and international validation.

Cluster analysis identified several relatively independent but internally cohesive thematic groups. Nodes such as “Deep Learning (DL),” “ultrasound,” “TIRADS,” and “computer-aided diagnosis” formed a technology-oriented cluster centered on intelligent imaging diagnosis, indicating that DL-based ultrasound image recognition and risk stratification remain dominant research directions. In parallel, pathology-related nodes, including “thyroid carcinoma”, “papillary”, “lymph node metastasis”, and “prognosis”, constituted another major cluster, suggesting that the field was shifting from simple image classification to clinical prognosis assessment and metastasis-risk prediction. In addition, nodes such as “feature extraction”, “neural network”, and “training” formed a methodological support layer, highlighting the foundational role of algorithm optimization and model training in advancing this research field.

### 3.7 Distribution Landscape of Journals in this Field

The visualization of journal distribution revealed a multidisciplinary publication ecosystem and thematic connections in AI research on thyroid space-occupying lesion diagnosis, from the perspective of knowledge dissemination and academic communication.

**Fig. (8):**
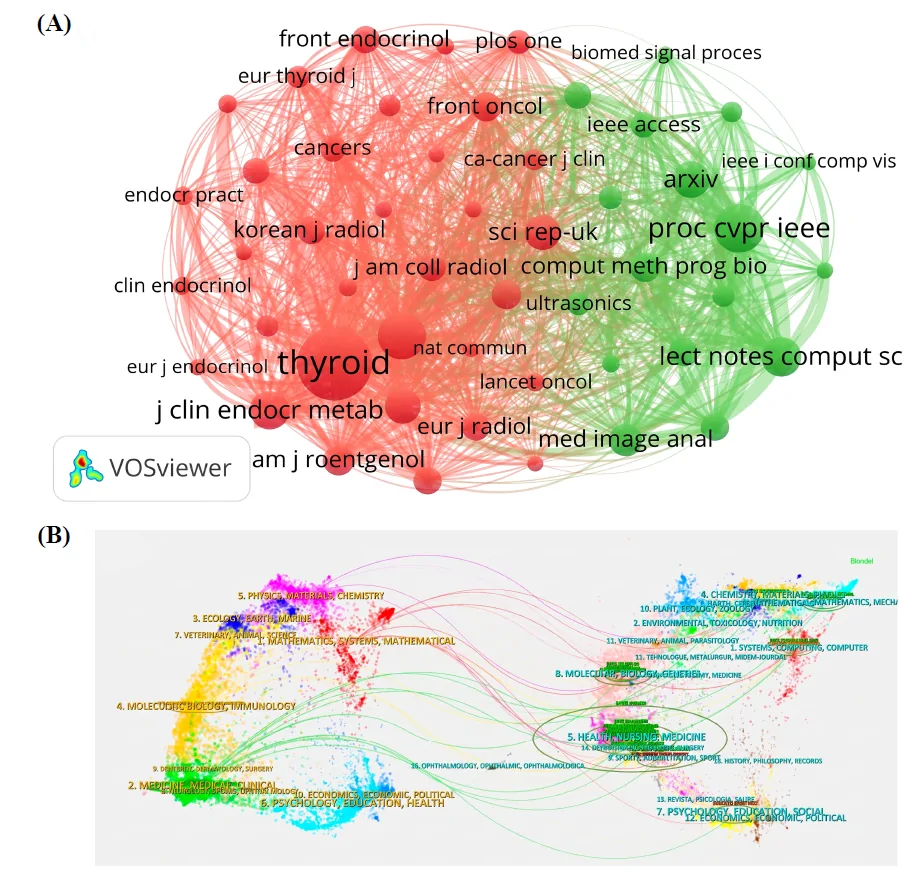
Journal visualization. This figure includes (**A**) Journal co-occurrence network map generated by VOSviewer, (**B**) Journal co-citation knowledge map (with citation count annotation).

The journal co-occurrence network was constructed using VOSviewer (Fig. **8A**). A total of 4702 journals were identified. When the publication threshold was set to 101, 50 journals met the criterion. In the network, nodes represent journals, and node size corresponds to the publication activity in this field. The links between nodes indicate thematic associations among journals. The color clustering distinguished four major journal clusters, each corresponding to a different disciplinary focus. Cluster 1 was the specialized endocrinology journal cluster, shown in green, with “Frontiers in Endocrinology”, “Thyroid”, “European Journal of Endocrinology”, and “Journal of Clinical Endocrinology and Metabolism” as the core journals. This cluster focused on the endocrinological characteristics, clinical diagnostic and therapeutic standards, and mechanisms of hormone regulation in thyroid space-occupying lesions, thereby providing clinical definitions and pathophysiological foundations for AI-related research. Cluster 2 was the comprehensive oncology and interdisciplinary journal cluster, shown in red, represented by “Frontiers in Oncology”, “Cancers”, and “Lancet Oncology”. This cluster emphasized the oncological characteristics of thyroid cancer, including cancer diagnosis, treatment, and prognosis, and also included interdisciplinary studies from medical imaging, bioinformatics, and AI. Therefore, it served as an important publication platform linking clinical oncology with technological innovations. The journal co-citation knowledge map revealed disciplinary intersection knowledge flow pathways in AI-related thyroid disease research (Fig. **8B**). Medical imaging journals, including “European Radiology” and “Quantitative Imaging in Medicine and Surgery”, were positioned close to endocrinology-related journals, such as “Thyroid” and “Frontiers in Endocrinology”. This distribution indicates the close integration of imaging research with clinical endocrinology, which forms the basis for technology-supported clinical diagnosis. In addition, the overlap between engineering and computer science journals, such as “IEEE ACCESS”, and medical imaging journals reflected the interaction between AI algorithms and imaging research, forming a knowledge bridge for AI-enabled thyroid disease diagnosis. Table **5** presents the most active journals according to their publication output, citation count, H-index, and total link strength. “Frontiers in Endocrinology” ranked first with 29 publications, indicating a strong representation of specialized clinical journals in this field. In terms of influence, “European Radiology” had 16 publications, which was not the highest output, but achieved 585 total citations and an H-index of 9, reflecting the strong single-article influence and academic recognition, consistent with its authoritative position in radiology. Most active journals were classified as JCR Q1 or Q2, indicating the generally high quality of research in this field. Notably, “Quantitative Imaging in Medicine and Surgery” also showed strong performance in publication output, with 15 articles, and in H-index, with a value of 10, highlighting the close relevance of quantitative imaging to this research area.

Overall, the journal ecosystem in this field has formed a relatively complete chain of knowledge circulation and collaboration. Clinical questions were mainly raised within the endocrinology and oncology journal clusters; imaging data support was provided by the radiology cluster; algorithmic advances were developed through engineering, technology, and interdisciplinary journal clusters; and clinical value was assessed through clinically oriented journals. This structure demonstrates the typical characteristics of an emerging interdisciplinary field.

### 3.8 Analysis of Funding Agencies

Research funding is a key driver of development in frontier scientific fields. According to the number of funded projects, the top 10 funding agencies supporting research on artificial intelligence-assisted diagnosis of thyroid space-occupying lesions were identified (Table **6**). Among them, the National Natural Science Foundation of China (NSFC) showed a dominant position, supporting 160 projects and accounting for nearly 50% of the total projects funded by the top 10 agencies. This pattern reflected China’s strong national-level investment in the integration of artificial intelligence, medical imaging, and precision medicine. Accordingly, national funding from China has provided substantial support for scientific output in this field. Local and ministerial funding bodies in China also played an important role. The Natural Science Foundation of Zhejiang Province, the Natural Science Foundation of Shandong Province, and the Science and Technology Commission of Shanghai Municipality (STCSM) were all listed among the top 10 funding agencies. This distribution indicates that research activity has been supported by a combined funding structure involving national-level leadership and local governmental participation, which has promoted the development and regional diffusion of research in areas with strong medical resources. Internationally, the National Research Foundation of Korea ranked second, with 26 funded projects, confirming South Korea as an important contributor to this field and corresponding to the strong performance of Korean institutions such as Yonsei University. In the United States, funding support was mainly provided by the National Institutes of Health (NIH), the United States Department of Health and Human Services, and its affiliated National Cancer Institute (NCI), reflecting the role of the national health research system in supporting advanced interdisciplinary research. Overall, the funding landscape for AI-assisted thyroid diagnosis showed a clear pattern, with China leading, followed by South Korea and the US. This multidimensional funding system, centered on Chinese national funding, supplemented by local governmental support and strengthened by international participation, can provide an important foundation for sustained technological innovation and clinical translation in this field.

### 3.9 Clinical Research Progress Analysis based on PubMed

Using a medical subject heading-based search strategy, 831 documents were retrieved from the PubMed database. Keyword clustering analysis (**Supplementary Material 3**) classified these studies into two main research directions. The first direction concerned the construction of technical systems and methodological innovations for artificial intelligence in clinical medicine. This direction focused on model optimization in “deep learning” and “computer-aided diagnosis”, as well as the medical image feature-extraction capability of “convolutional neural networks”, thereby clarifying the core supporting role of AI in intelligent clinical disease diagnosis. The second direction focused on the clinical application value of AI-assisted diagnostic technologies in early screening and benign/malignant differentiation of “thyroid nodules” and “thyroid cancer”.

## 4 DISCUSSION

### 4.1 General Information

This study presents a systematic bibliometric analysis of research on AI applications in thyroid lesion diagnosis to clarify its current status, developmental trajectory, and structural characteristics. Publication trend analysis showed that over the past two decades (2006–2025), this field progressed from a stage of technological exploration (2006–2018) to rapid growth (2019–2021) and then to accelerated mainstream development (2022–2025). In particular, both publication output and citation frequency increased rapidly, with citation growth exceeding publication growth. This pattern suggests that the field has moved from an emerging innovation stage towards early growth, while its research findings have gained increasing academic recognition and practical application.

At the national and institutional levels, China contributed the largest publication output, with 371 papers, reflecting strong research mobilization and resource investment. However, its average citations per paper (16.57) were lower than those of the United States and South Korea (23.60), indicating that further improvement is still needed in research innovation and international academic influence, despite its high output scale. The US contributed 81 papers and showed a strong academic influence, as reflected by its higher average citations per paper, H-index of 26, and international cooperation link strength of 86, supporting its central role in the global collaboration network. This pattern was further reflected in the institutional cooperation network, which contained three well-defined research clusters: a clinical problem-driven cluster centered on leading Chinese medical universities and affiliated hospitals, an interdisciplinary algorithm innovation cluster represented by the Chinese Academy of Sciences, and an international multicenter validation cluster linking high-level medical centers in China and South Korea. These clusters were not isolated from one another but formed a complementary functional chain involving clinical problem identification, methodological model development, and multicenter clinical validation, thereby constituting the main structure of knowledge production and translation in this field. The consistency between the macroscopic collaboration pattern and the microscopic keyword structure was further revealed by keyword analysis. The three technical pillars identified through keyword co-occurrence and clustering analysis, namely DL-based thyroid nodule detection and classification, multimodal feature fusion and model optimization, and clinical translation with risk stratification, corresponded closely to the clinical-driven, algorithm innovation, and international validation functions of the institutional clusters, respectively. For instance, the first technical pillar centered on “classification”, “cancer,” and “deep learning” was strongly supported by research outputs from the clinical problem-driven cluster. The second pillar, represented by “feature fusion” and “attention mechanism,” aligned closely with the research mission of the algorithm innovation cluster. The emerging direction of “risk stratification” requires diverse clinical cohorts and standardized evaluation procedures, which can be mainly provided by an international multicenter validation cluster. These findings indicate a close structural relationship between the national and institutional research layout and the evolution of technical hotspots. China’s large-scale output can provide advantages in data resources and application scenarios for technology validation, whereas the strengths of the US and South Korea in high-impact research and international collaboration support methodological innovation and integration with clinical guidelines.

Overall, AI research in thyroid lesion diagnosis has developed into a globally structured, functionally differentiated, and dynamically evolving research system. This system is driven by internal technological logic, particularly DL, and is also shaped by external factors, including national research capacity, institutional collaboration models, and clinical translation pathways. Understanding these multilevel and interrelated development patterns is important for assessing the current state of the field, anticipating future trends, and improving research resource allocation.

With the broader application of AI in medical diagnosis, disease management, and follow-up, AI-based thyroid disease diagnosis and management have gained increasing importance. Table **7** summarizes six representative AI models covering thyroid ultrasound image analysis, biopsy pathology-assisted diagnosis, rare subtype detection, multimodal data fusion, and primary care screening. By automatically extracting nodule features, integrating the TI-RADS grading system, generating synthetic images, and detecting report errors, AI may improve diagnostic accuracy, accelerate processing, and reduce unnecessary biopsies and biopsy-related disputes [46-54]. Based on a systematic literature review and clinical data comparison, Table **8** evaluates the AI performance in medical diagnosis across five dimensions: diagnostic accuracy, diagnostic efficiency, diagnostic consistency, clinical decision-making, and cost. The results indicated that AI showed advantages over conventional diagnostic approaches in several key aspects, including diagnostic accuracy, processing efficiency, result consistency, and medical cost reduction. Overall, these AI tools may optimize clinical workflows and provide technical support for primary medical care and health management, suggesting a broad potential for improving early diagnosis and diagnostic consistency in thyroid disease [55-75].

### 4.2 Research Hotspots and Frontiers

Keyword co-occurrence and clustering analyses delineated the knowledge structure and research frontiers of AI-assisted diagnosis of thyroid space-occupying lesions. Based on high-frequency keywords and cluster labels, the current research landscape can be divided into three major directions:

#### 4.2.1 Cluster 1: “Thyroid Nodule Detection and Classification Based on Deep Learning”

Cluster 1 was mainly characterized by keywords such as “classification”, “cancer”, “deep learning”, and “ultrasound image”. This research direction focused on the application of DL architectures, particularly CNNs [76], for automated detection and benign/malignant classification of thyroid nodules in ultrasound images [77]. Its primary purpose is to improve diagnostic objectivity, consistency, and efficiency. Evidence from previous studies suggests that AI-based CAD systems can achieve, and in some datasets even surpass, the diagnostic performance of experienced radiologists [78, 79]. Such systems may be particularly useful for challenging cases, including small cancers and atypical nodules [80, 81]. Therefore, current work in this cluster is concentrated on model architecture optimization, refinement of training strategies, and validation of generalizability across multicenter and multi-device datasets.

#### 4.2.2 Cluster 2: “Multi-modal Feature Fusion and Model Optimization”

Cluster 2 was represented by keywords including “feature fusion”, “computational model”, and “attention mechanism”. The investigation in this direction aimed to enhance model representation and robustness by integrating multilevel and multimodal features. Advanced techniques, especially attention mechanisms, allow models to focus more effectively on key imaging regions [82] and improve the interpretability of AI-assisted decision-making, thereby offering clinicians more intuitive and reliable diagnostic support. TL has also been widely adopted to address the limited availability of labeled medical imaging data [83], which can accelerate model convergence and improve performance in thyroid diagnostic tasks.

#### 4.2.3 Cluster 3: “Clinical Translation and Risk Stratification Application”

The emergence of keywords such as “semantic segmentation” and “risk stratification” in Cluster 3 indicated that research has moved beyond simple benign/malignant discrimination towards more clinically oriented applications. Semantic segmentation can achieve pixel-level delineation of nodule regions, providing a basis for subsequent quantitative analysis and morphological evaluation. Risk stratification represents an important translational direction, as it seeks to connect AI outputs with clinical guidelines, including ATA and ACR TI-RADS, to support individualized risk estimation [84] and assist clinicians in formulating more precise follow-up and treatment strategies. This trend reflects the gradual transition of AI from assisted lesion detection to clinically actionable decision support.

#### 4.2.4 Deeper Analysis of Research Trends

The integration of publication trends, keyword evolution, and collaboration network structures revealed three major research trends. First, the field has shifted from technical validation to clinical integration. Whereas early studies mainly emphasized model construction and performance comparison, the recent rise of keywords such as “risk stratification” and “guidelines” suggests increasing efforts to embed AI into clinical workflows and align it with established diagnostic guidelines. Second, the field has expanded from unimodal imaging analysis to multimodal fusion. The growing prominence of “feature fusion”, “attention mechanism”, and “multimodal” indicates that research is moving beyond ultrasound images alone and is increasingly incorporating clinical text, pathology, genomic data, and other information sources to construct more comprehensive diagnostic models. Third, research activity is progressing from single-center studies to global multicenter collaborations. Strengthening collaboration among China, the US, and South Korea, particularly between high-level Chinese and Korean medical institutions, reflects the increasing importance of multicenter validation for improving model generalizability and facilitating clinical adoption. These trends indicate that AI is evolving from a supplementary diagnostic tool to an integrated decision-support system with a more active role in clinical decision-making.

#### 4.2.5 Methodological Evolution of AI Techniques

Beyond the clustering of research topics, the bibliometric results also showed a clear methodological evolution in AI techniques. Before 2017, traditional ML methods based on hand-crafted features, including texture analysis and morphological descriptors, were commonly used for classification, with support vector machines serving as a representative method [45]. More recently, DL has become the dominant approach. CNN-based architectures, including VGG, ResNet, and Inception, have enabled end-to-end learning from raw image data by automatically extracting features, thereby improving diagnostic accuracy. This shift was reflected in the surge in the keyword “convolutional neural network” during 2017–2018. Research attention has subsequently shifted towards model robustness and clinical utility. Attention mechanisms have been introduced to improve interpretability by highlighting the most relevant regions in ultrasound images, making AI-based decisions more transparent to clinicians [82]. To address the shortage of labeled medical imaging data, TL [30, 31] and self-supervised learning [18] enable models to be pre-trained on large general datasets and then fine-tuned on smaller thyroid imaging datasets. Emerging multimodal learning models, such as “ThyGPT” [53] (Table **7**), represent the current frontier by integrating ultrasound images, clinical reports, genomic data, and other information sources to support more holistic and personalized diagnosis. This progression from hand-crafted features to deep learning and then to interpretable multimodal AI reflects the rapid methodological maturation of the field and its increasing integration with clinical practice.

#### 4.2.6 Relevant Research in the Field of Medical Image Analysis

Broader advances in medical image analysis also provide important methodological references for AI-assisted thyroid lesion diagnosis. For example, the review and meta-analysis by Aggarwal *et al*. reported that DL models can achieve the diagnostic accuracy comparable to, or higher than, that of human experts across multiple medical imaging tasks, providing evidence for the clinical translation of AI tools [55]. Yamashita *et al.* developed a DL-based risk stratification system using ultrasound cine-clip images and showed that temporal information may help reduce false-positive biopsy recommendations [36]. Tang *et al.* proposed a dual-stream attention neural network for benign/malignant diagnosis of thyroid nodules, demonstrating that the integration of texture and shape features can improve model performance and interpretability [37]. These findings from the broader medical image analysis literature support the bibliometric results of this study and indicate potential future directions, including dynamic image analysis and attention-guided feature learning. Incorporating these cross-disciplinary insights may promote the development and clinical adoption of AI in thyroid disease diagnosis.

### 4.3 Current Challenges and Future Development

Through sustained efforts in this field, AI has progressed from an exploratory concept to an active research direction for the diagnosis of thyroid mass lesions. Research topics have become increasingly diverse, and technological advances continue to emerge [85]. However, the bibliometric findings also indicate that several developmental bottlenecks remain, along with considerable room for further improvement. Based on the current evidence, future research should be deepened and expanded in the following three directions:

(1) From Data Islands to Generalizable Foundation Models: Current AI models remain largely dependent on retrospective and single-center datasets, which limit their robustness and transferability to new clinical settings. A key future direction is the construction of large-scale, prospective, multicenter thyroid imaging databases based on standardized protocols for image acquisition and annotation. Such efforts should extend beyond existing bilateral collaborations, including China-US and China-South Korea partnerships, and develop into a genuinely global research consortium. The aim should not be limited to data aggregation. Conversely, it should include the development and validation of foundational models for thyroid ultrasound. When pre-trained on heterogeneous global datasets, these models may achieve stronger generalizability and allow reliable fine-tuning for specific clinical tasks or institutional environments with limited local data. This transition from dataset-specific models to shared and generalizable AI foundations is essential for broader clinical applications.

(2) From “Black Box” to Actionable Clinical Decision Support: Although diagnostic accuracy is essential, limited interpretability remains a major barrier to clinical trust in AI systems. Therefore, future studies should prioritize Explainable AI (XAI) [86, 87], which can provide intuitive visual and textual explanations for model predictions, including highlighting key nodule features such as microcalcifications or irregular margins. A further critical step is the transition from offline validation to prospective Randomized Controlled Trials (RCTs), in which XAI tools are embedded into real-world clinical workflows. These trials should evaluate clinically meaningful outcomes, including reductions in unnecessary fine-needle aspiration biopsies, improvements in inter-rater agreement among clinicians, and the cost-effectiveness of AI-assisted diagnostic pathways compared with standard care. The objective is to determine whether AI tools can not only maintain high diagnostic accuracy but also produce measurable improvements in patient management and healthcare efficiency.

(3) From Image Analysis to Holistic, Multimodal Precision Diagnosis: Ultrasound image analysis alone provides an incomplete representation of the complex diagnostic process. Future progress in this field will depend on the deep integration of multiple data modalities, including ultrasound combined with elastography or contrast-enhanced ultrasound [88, 89], clinical parameters such as TSH levels and patient age, molecular biomarkers such as BRAF and TERT mutations [90-92], and textual information extracted from pathology reports. This integration requires advanced multimodal fusion algorithms capable of processing heterogeneous data types. Through a holistic multimodal framework, AI may progress beyond simple benign/malignant classification toward more advanced clinical tasks, including individualized recurrence-risk stratification, prediction of lymph node metastasis, and support for surgical or follow-up strategy selection, thereby contributing to precision medicine in thyroid disease diagnosis.

### 4.4 Implications for Clinical Guidelines and Practice

This bibliometric analysis provides relevant evidence for the future incorporation of AI into clinical guidelines for thyroid lesion diagnosis. Current guidelines, including the American Thyroid Association (ATA) guidelines and the American College of Radiology (ACR) TI-RADS [93, 94], rely mainly on ultrasound features and clinical risk factors, whereas AI-based diagnostic tools have not yet received formal emphasis. The findings of this study indicate that AI research is gradually shifting from basic classification tasks to clinical risk stratification, as reflected by keywords such as “risk stratification”, “association guidelines”, and “computer-assisted diagnosis”. This shift suggests a closer alignment between AI model development and clinical management requirements. Several findings support the potential inclusion of AI in future guideline updates. First, the high diagnostic accuracy reported for AI models in large-scale studies (Table **7**) suggests that AI may serve as an effective supplement to human interpretation, particularly in primary care settings where access to experienced radiologists may be limited. Second, multimodal AI systems that integrate ultrasound imaging with genomic data may provide more individualized risk assessments than existing category-based approaches. Finally, increasing evidence from multicenter validation studies, including China-South Korea collaboration clusters, may provide external validation to support the future integration of AI into clinical guidelines.

## 5 STRENGTHS AND LIMITATIONS

This study is the first systematic bibliometric analysis of global research on artificial intelligence-assisted diagnosis of thyroid space-occupying lesions. In contrast to traditional reviews, which may be constrained by authors’ subjective interpretations and the scope of the selected literature, this study used visualization tools, including VOSviewer and CiteSpace, to conduct quantitative mining, dynamic trend tracking, and collaboration network visualization for publications from 2006 to 2025. Through this data-driven approach, the limitations of conventional narrative synthesis were reduced, while latent knowledge connections and developmental patterns in the field were revealed more clearly. This study provides a systematic, objective, and forward-looking basis for research evaluation and strategic planning in this area.

Based on refined search strategies and strict data-cleaning procedures, 652 core documents published over the past two decades were included. Their developmental trajectories, national and institutional contributions, collaboration patterns, research hotspots, and frontier directions were comprehensively examined. By identifying the scientific collaboration structure, including the dual-core pattern centered on China and the United States, and clarifying the technological evolution from basic classification toward risk stratification, this study provides a reference framework for research teams engaged in AI-assisted thyroid diagnosis. These findings may also support a more rational allocation of research resources and promote clinical translation and application-oriented innovation in this field.

Nevertheless, this study has several limitations. First, the dataset was derived only from WoSCC. Although this database ensures academic quality and provides standardized bibliometric records, it may not fully capture all relevant studies, particularly publications from regional journals, non-English literature, and important conference proceedings. Consequently, the completeness of the global research landscape may be affected. Second, bibliometric analyses are inherently subject to certain biases. Author self-citation and the Matthew effect associated with well-known teams or high-impact journals may overrepresent specific researchers or publications, while valuable but less visible contributions may be underestimated. Methodological limitations also exist for the analytical tools. CiteSpace burst detection may overemphasize short-term hotspots and underrepresent research directions with a sustained influence. VOSviewer clustering depends on preset similarity thresholds, which may isolate or overlook marginal knowledge flows across disciplines and topics. These factors may affect the accuracy of trend interpretation to some extent [95, 96]. Finally, this study primarily analyzed and projected research trends based on historical literature. Because frontier technologies evolve rapidly, breakthrough algorithms, major clinical validation results, and sudden changes in public health policy may alter the field's developmental trajectory, making predictions based solely on historical evidence uncertain.

Overall, these limitations do not materially affect the core findings or main conclusions of this study, although they may slightly reduce the precision of several trend descriptions. They also indicate directions for future research. Subsequent studies could integrate multiple databases, such as Scopus and PubMed, with patent data and clinical trial registration information to construct a more comprehensive analytical map. Future work could also combine DL with other emerging technologies to deepen semantic mining of full-text content and introduce additional dimensions of academic influence evaluation, thereby presenting the knowledge system and innovation dynamics of this field more comprehensively and dynamically.

## CONCLUSION

Through a systematic bibliometric analysis, this study characterized the research landscape and developmental dynamics of artificial intelligence in the diagnosis of thyroid space-occupying lesions from 2006 to 2025. The findings showed that this field has progressed from a fragmented stage of technological exploration to rapid expansion and, more recently, to a mainstream direction of research and clinical innovations. Globally, research output has become concentrated mainly in China, the US, and South Korea. China contributed the largest number of publications, whereas the US showed a stronger overall influence on research impact and international collaboration. Institutional and author collaboration networks revealed an organic division of labor involving clinical problem-driven research, basic algorithm innovation, and international multicenter validation, thereby forming a research pipeline from theoretical development to clinical application. Keyword co-occurrence, clustering, and burst analyses further identified three fundamental knowledge structures and frontier directions: (1) DL-based thyroid nodule detection and classification, in which convolutional neural networks and related architectures are used for end-to-end benign/malignant discrimination on ultrasound images; (2) multimodal feature fusion and model optimization, in which attention mechanisms and other advanced techniques are applied to improve model expressiveness, robustness, and interpretability; and (3) clinical translation and risk stratification, through which research is moving from simple lesion classification toward more sophisticated risk assessment and management for accurate clinical decision-making. Although this field still faces challenges, including the need for China to enhance its international academic influence while maintaining high output and the need to close cross-cluster cooperation gaps in the global collaboration network, future research should continue to promote deeper cross-regional and cross-disciplinary collaboration between algorithm experts and clinicians. Research paradigms should also advance towards multimodality, interpretability, and prospective clinical validation by integrating multidimensional data, including ultrasound imaging, clinical pathology, and genomics, to evaluate model generalizability and clinical performance. In addition, emerging technologies, such as large language models, may be explored for report generation and causal inference in prognostic prediction, thereby supporting further innovations in this field. Overall, this study provides a structured overview of the knowledge landscape, collaboration patterns, and future directions of AI-assisted thyroid lesion diagnosis.

## Figures and Tables

**Table 1:** Top 5 countries in terms of publication volume.

**Country**	**Documents**	**Citations**	**Average Citations**	**H-index**	**Total Link Strength**
China	371	6149	16.57	36	71
United States	109	2987	27.4	26	86
South Korea	58	1369	23.6	19	31
Italy	26	618	23.77	14	26
India	21	176	8.38	7	13

**Table 2:** Top 10 institutions in terms of publication volume.

**Institution**	**Documents**	**Citations**	**Average Citations**	**H-index**	**Total Link Strength**
Shanghai Jiao Tong University	40	871	21.78	15	36
Chinese Academy of Sciences	37	536	14.49	14	85
Zhejiang University	24	634	26.42	10	55
Sichuan University	21	332	15.81	10	13
Tianjin Medical University	19	309	16.26	9	16
Yonsei University	19	330	17.37	9	3
Zhejiang Cancer Hospital	19	338	17.79	9	35
Fudan University	18	429	23.83	9	4
Southern Medical University, China	17	496	29.18	10	19
Sun Yat Sen University	17	633	37.24	13	11

**Table 3:** Top 10 authors in terms of publication volume.

**Authors**	**Documents**	**Citations**	**H-index**	**Affiliations**	**Country**
Xu, Dong	18	317	10	Chinese Academy of Sciences	China
Yao, Jincao	16	118	9	Chinese Academy of Sciences	China
Wei, Xi	14	280	11	Tianjin Medical University	China
Kwak, Jin Young	11	232	8	Yonsei University	Korea
Chen, Chen	10	57	8	Chinese Academy of Sciences	China
Lee, Eunjung	9	160	6	Yonsei University	Korea
Xu, Lei	9	93	8	Karolinska Institutet	Sweden
Ha, Eun Ju	8	271	8	Ajou University	Korea
Luo, Yukun	8	87	7	Southern Medical University - China	China
Yoon, Jung Hyun	8	218	8	Yonsei University College of Medicine	Korea

**Table 4:** Top 10 key words co-occurrence in terms of publication volume.

**Key word**	**Occurrences**	**Total Link Strength**
Accuracy	217	192
Artificial intelligence	179	669
Association	171	146
Association guidelines	161	296
Benign	142	424
Bethesda system	132	108
Cancer	123	1168
Carcinoma	121	341
Classification	117	679
Computer-aided diagnosis	100	361

**Table 5:** Top 10 journals in terms of document volume.

**Journal**	**Citation**	**Total Link Strength**	**H-index**	**Document**	**JCR** **Ranking**	**Impact factor (2024)**
Frontiers in Endocrinology	153	6079	8	29	Q1	4.6
Frontiers in Oncology	231	8694	9	28	Q2	3.3
IEEE Access	300	5641	8	22	Q2	3.6
Scientific Reports	236	6362	8	19	Q2	4.4
Thyroid	585	27711	9	16	Q1	6.7
Endocrine	83	6389	4	15	Q3	2.9
European Radiology	266	11154	10	15	Q1	4.7
Diagnostics	86	3393	5	14	Q1	3.3
Quantitative Imaging in Medicine and Surgery	92	2382	6	14	Q2	2.3
Cancer	189	6362	8	13	Q2	4.4

**Table 6:** Top 10 funding agencies in terms of publication volume.

**Rank**	**Funding Agencies**	**Count**	**Rank**	**Funding Agencies**	**Count**
1	National Natural Science Foundation of China (NSFC)	160	6	National Key Research Development Program of China	17
2	National Research Foundation of Korea	26	7	Ministry of Science, ICT, and MSIT, Republic of Korea	15
3	National Institutes of Health Nih Usa	22	8	Natural Science Foundation of Shandong Province	12
4	United States Department of Health and Human Services	22	9	Nih National Cancer Institute Nci	12
5	Natural Science Foundation of Zhejiang Province	19	10	Science Technology Commission of Shanghai Municipality Stcsm	12

**Table 7:** Common AI applications in thyroid disorders.

**No.**	**Name of AI Model**	**Application Scenarios**	**Specific Functions**	**Effects**	**References**
1	ThyNet	Thyroid ultrasound image diagnosis	Automatically extract key features (*e.g*., nodule edges, calcification, morphology) and integrate the TI-RADS classification system for risk stratification	Doctor’s diagnostic accuracy increased from 83.7% to 87.5%; proportion of unnecessary punctures decreased from 61.9% to 35.2%	[46-48]
2	S-Detect System	Thyroid ultrasound image diagnosis	Automatically complete TI-RADS classification of thyroid nodules *via* deep learning algorithms to assist primary care doctors	Resident physicians’ diagnostic specificity increased from 42.9% to 76.2%; reduced risks of missed diagnosis and overdiagnosis	[49,50]
3	ThyroPower	Pathological diagnosis of thyroid needle biopsy	Extract morphological features of malignant cells based on digital pathological image analysis; assist in judging “indeterminate” pathological cases	Junior pathologists’ diagnostic accuracy increased from 87.7% to 94.8%; reduced discrepancies in pathological diagnosis	[51]
4	Tiger Model	Thyroid ultrasound diagnosis (for rare subtypes)	Integrate disease knowledge bases to generate text prompts; guide diffusion models to produce synthetic ultrasound images of rare subtypes (*e.g*., FTC, MTC) for small-sample data supplementation	AUROC for benign-malignant classification of FTC and MTC increased by 14.64% and 9.45%, respectively; AUC increased by ≥0.07 in small-sample scenarios	[52]
5	ThyGPT	Diagnosis and management of thyroid nodules	Achieve risk assessment, classification, and report error detection *via* a multi-head self-attention mechanism (multimodal data fusion: ultrasound images + text reports)	Radiologists’ evaluated AUC increased from 0.805 to 0.908; biopsy rate decreased to 23.3%; error detection rate = 90.5%; report processing time = 0.031 seconds	[53]
6	Thyroid AI Ultrasound Assisted Diagnosis System	Routine physical examination screening, clinical auxiliary diagnosis, and postoperative follow-up monitoring	Automatically locate nodules; analyze indicators (*e.g*., aspect ratio, boundary, echo); output standardized TI-RADS classification evaluation	Improved early diagnosis rate of thyroid diseases; optimized primary medical service quality; enhanced residents’ health management efficiency	[54]

**Table 8:** AI diagnosis advantages summary.

**No.**	**Category**	**Evaluation Indicators**	**AI Diagnosis Performance**	**Conventional Diagnosis Performance**	**Explanation of AI Advantages**	**References**
1	Diagnostic Accuracy	Overall accuracy	87-95%	58-86%	AI’s accuracy is equal to or exceeds that of doctors in most medical image diagnoses, especially in complex cases	[55-57]
		Detection of small lesions	Missed diagnosis rate of small nodules reduced to 3%	Missed diagnosis rate ≈ 15%	The detection rate of small nodules (≤5mm) increased by 20%, significantly improved early detection rate	[58,59]
		Diagnosis of difficult cases	Differential diagnosis accuracy = 78.3%	Low comprehensiveness of differential diagnosis	Provides more comprehensive differential diagnosis in complex cases; reduces missed diagnosis	[60,61]
		Performance in primary care	Accuracy = 90-92%	Greatly affected by doctors’ experience (≈70-80%)	Compensates for primary care doctors’ lack of experience; brings diagnostic level close to that of top tertiary hospitals	[62,63]
2	Diagnostic Efficiency	Single case diagnosis time	Average 0.744 minutes (CT)	Average 3.623 minutes	AI speeds up diagnosis by ~5x; especially suitable for emergency and large-scale screening	[64]
		Batch processing capability	Analyzes thousands of images simultaneously	Doctors read ≈200 images per day on average	AI’s efficiency improved by >10x; greatly shortens waiting time	[65]
		Follow-up evaluation speed	Automatic registration and comparison; efficiency improved by 8x	Manual comparison is time-consuming and labor-intensive	Significantly enhances the efficiency of chronic disease management and treatment effect evaluation	[66]
		Report generation	Generates structured reports in real time	Requires manual organization by doctors	Report efficiency improved by 70%; reduces paperwork	[67]
3	Diagnostic Consistency	Inter-doctor differences	Consistency score > 95%	Consistency ≈ 60-80%	Eliminates diagnostic deviations caused by doctor fatigue and experience gaps	[68]
		Consistency of repeated examinations	Consistent results for multiple diagnoses of the same case	Diagnosis result differences can reach ≈20% across different time points	Ensures continuity of treatment plans; reduces repeated examinations	[69]
		The gap between primary care and expert diagnosis	Primary hospitals’ accuracy increased to ≈90%	The gap between primary hospitals and top tertiary hospitals ≈20%	Popularizes high-quality medical resources; narrows urban-rural medical gaps	[70]
4	Auxiliary Clinical Decision-Making	Broaden diagnostic thinking	Comprehensiveness score increased by ≈20%	-	Provides differential diagnoses that doctors may overlook	[60]
		Decision support	Auxiliary diagnosis accuracy increased by 6.8-9.8%	-	Especially, 8.9% improvement in actionable decisions	[71]
		Risk stratification	Accurately assesses disease risk and predicts prognosis	-	Assists in treatment plan selection	[72]
		Multimodal fusion	Integrates multi-source data; diagnostic accuracy increased by ≈20%	-	Fuses imaging, pathology, and genomics data	[73-74]
5	Cost-Effectiveness	Labor costs	Reduces diagnostic physicians’ workload by 50-70%	Requires a large number of professional physicians	Frees up doctors’ time to focus on complex decisions and patient communication	[75]
		Misdiagnosis costs	Misdiagnosis rate reduced by 20-40%	High misdiagnosis rate (especially for rare diseases)	Avoids repeated examinations and incorrect treatment; saves medical expenses	[69]
		Screening costs	Large-scale screening costs reduced by 60%	High cost and limited coverage	Improves early disease detection rate; reduces later treatment costs	[59]
		Equipment investment	One-time high investment; long-term cost reduction	Continuous human investment; increasing costs	In the long run, AI systems are more cost-effective (especially in high-load scenarios)	[75]

## Data Availability

In this study, the data involved are not sensitive in nature. They were sourced from the public domain and are therefore accessible and free from confidentiality concerns. This study utilized a series of analytical tools, such as Excel (Microsoft Office 2021), VOSviewer 1.6.20.0, CiteSpace 6.4.R1, Microsoft Excel 2019, and RStudio 2024.12.1, to conduct in-depth analyses of the acquired data, thereby generating the data results reported in this paper. All data used in this study have been fully incorporated into the main body of the article or supplementary materials. It should be noted that no custom code was generated in this study. If there is a need to re-analyze the data reported in this paper or obtain additional information, an application can be made to the principal contact, and relevant information will be provided as required.

## LIST OF ABBREVIATIONS

AI: Artificial Intelligence
CAD: Computer-Aided Diagnosis
CNNs: Convolutional Neural Networks
FNAB: Fine-Needle Aspiration Biopsy
H-index: Hirsch index
PTC: Papillary Thyroid Cancer
WOSCC: Web of Science Core Collection
ACR: American College of Radiology
ATA: American Thyroid Association
RCTs: Randomized controlled trials
XAI: Explainable AI
ML: Machine learning
DL: Deep learning
US: The United States
NSFC: The National Natural Science Foundation of China
UK: The United Kingdom
NIH: The National Institutes of Health
NCI: National Cancer Institute
