# Supplementary material for: Dual-database Bibliometric Analysis Combined with Gephi-based Network Visualization of Artificial Intelligence Applications in the Identification and Diagnosis of Thyroid Space-occupying Lesions
Source: Curr Med Imaging. 2026 Jun 16;22:E15734056466850. doi: 10.2174/0115734056466850260610101215 (PMC13602106; doi:10.2174/0115734056466850260610101215)
Supplement: Supplementary file 1 [file CMIM-22-E15734056466850_SD1.pdf]

1875-6603/26 Send Orders for Reprints to reprints@benthamscience.net

Current Medical Imaging

Content list available at: https://benthamscience.com/journals/cmir

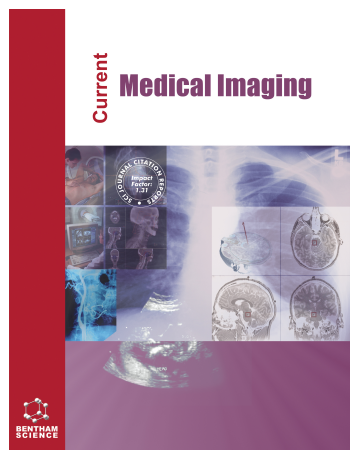

Supplementary Material

Dual-Database Bibliometric Analysis Combined with Gephi-Based Network Visualization of Artificial Intelligence Applications in the Identification and Diagnosis of Thyroid Space-Occupying Lesions

Shilin Wen[1,#], Weiqing Qian[1,#], Yuyang Su[1], Xiaohan Cao[1], Toutou Chen[1], Weidong Gong[2,*] and Xiaocan Lei[1,*]

[1]*Clinical Anatomy and Reproductive Medicine Application Institute, The First Affiliated Hospital, Hengyang Medical School, University of South China, Hengyang 421001, China*
[2]*Affiliated Hengyang Hospital of Hunan Normal University & Hengyang Central Hospital, Hengyang, 421001, Hunan, China*

WOS

2006-2025

#1 TS = ("transfer learning" OR "artificial intelligence" OR "AI" OR "neural network" OR "machine learning" OR "deep learning") 1049205

#2 TS = ("Detection" OR "Classification" OR "Diagnosis") 3749483

#3 TS = ("thyroid cancer" OR "thyroid nodule" OR "thyroid space-occupying lesion") 35336

#4 #1 and #2 and #3 and English (Languages) and Article or Review Article (Document Types) 674

After manual screening Excluded document types (n=19), Did not meet study design criteria(n=3) 652

PubMed

2006-2025

#1((((((((((((((((((((Artificial Intelligence[MeSH Terms]) OR (Artificial Intelligence[Title/Abstract])) OR (Intelligence, Artificial[Title/Abstract])) OR (Computer Reasoning[Title/Abstract])) OR (Reasoning, Computer[Title/Abstract])) OR (AI (Artificial Intelligence[Title/Abstract]))) OR (Machine Intelligence[Title/Abstract])) OR (Intelligence, Machine[Title/Abstract])) OR (Computational Intelligence[Title/Abstract])) OR (Intelligence, Computational[Title/Abstract])) OR (Computer Vision Systems[Title/Abstract])) OR (Computer Vision System[Title/Abstract])) OR (System, Computer Vision[Title/Abstract])) OR (Systems, Computer Vision[Title/Abstract])) OR (Vision System, Computer[Title/Abstract])) OR (Vision Systems, Computer[Title/Abstract])) OR (Knowledge Acquisition (Computer[Title/Abstract]))) OR (Acquisition, Knowledge (Computer[Title/Abstract]))) OR (Knowledge Representation (Computer[Title/Abstract]))) OR (Knowledge Representations (Computer[Title/Abstract]))) OR (Representation, Knowledge (Computer[Title/Abstract])) 357518

#2 (((((((((((((((Diagnosis[MeSH Terms]) OR (Diagnosis[Title/Abstract])) OR (Diagnoses[Title/Abstract])) OR (Diagnose[Title/Abstract])) OR (Diagnoses[Title/Abstract] AND Examinations[Title/Abstract])) OR (Diagnoses[Title/Abstract] AND Examination[Title/Abstract])) OR (Examination[Title/Abstract] AND Diagnoses[Title/Abstract])) OR (Examinations[Title/Abstract] AND Diagnoses[Title/Abstract])) OR (Antemortem Diagnosis[Title/Abstract])) OR (Antemortem Diagnoses[Title/Abstract])) OR (Diagnoses, Antemortem[Title/Abstract])) OR (Diagnosis, Antemortem[Title/Abstract])) OR (Postmortem Diagnosis[Title/Abstract])) OR (Diagnoses, Postmortem[Title/Abstract])) OR (Diagnosis, Postmortem[Title/Abstract])) OR (Postmortem Diagnoses[Title/Abstract]) 11322100

#3 ((((((((((((((((((Thyroid Neoplasms[MeSH Terms]) OR (Thyroid Neoplasms[Title/Abstract])) OR (Neoplasm, Thyroid[Title/Abstract])) OR (Thyroid Neoplasm[Title/Abstract])) OR (Neoplasms, Thyroid[Title/Abstract])) OR (Thyroid

DOI: 10.2174/0115734056466850260610101215, 2026, *22*, e15734056466850

Carcinoma[Title/Abstract])) OR (Carcinomas, Thyroid[Title/Abstract])) OR (Carcinoma, Thyroid[Title/Abstract])) OR (Thyroid Carcinomas[Title/Abstract])) OR (Cancer of the Thyroid[Title/Abstract])) OR (Cancer of Thyroid[Title/Abstract])) OR (Thyroid Cancers[Title/Abstract])) OR (Thyroid Cancer[Title/Abstract])) OR (Cancers, Thyroid[Title/Abstract])) OR (Cancer, Thyroid[Title/Abstract])) OR (Thyroid Adenoma[Title/Abstract])) OR (Adenomas, Thyroid[Title/Abstract])) OR (Adenoma, Thyroid[Title/Abstract])) OR (Thyroid Adenomas[Title/Abstract]) 86108

#4 #1 and #2 and #3 872

After manual screening Excluded document types (n=20), Did not meet study design criteria(n=21) 831

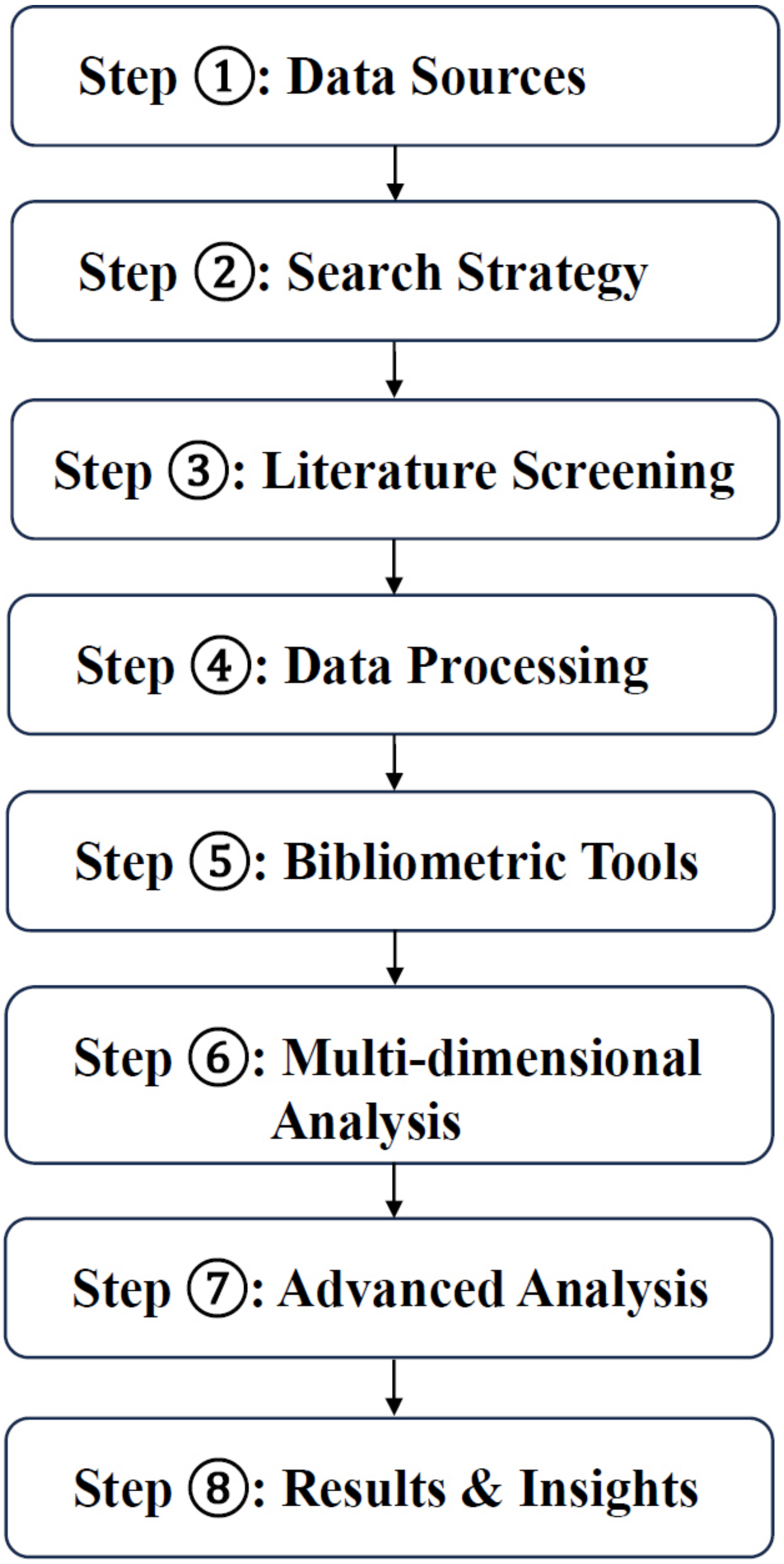

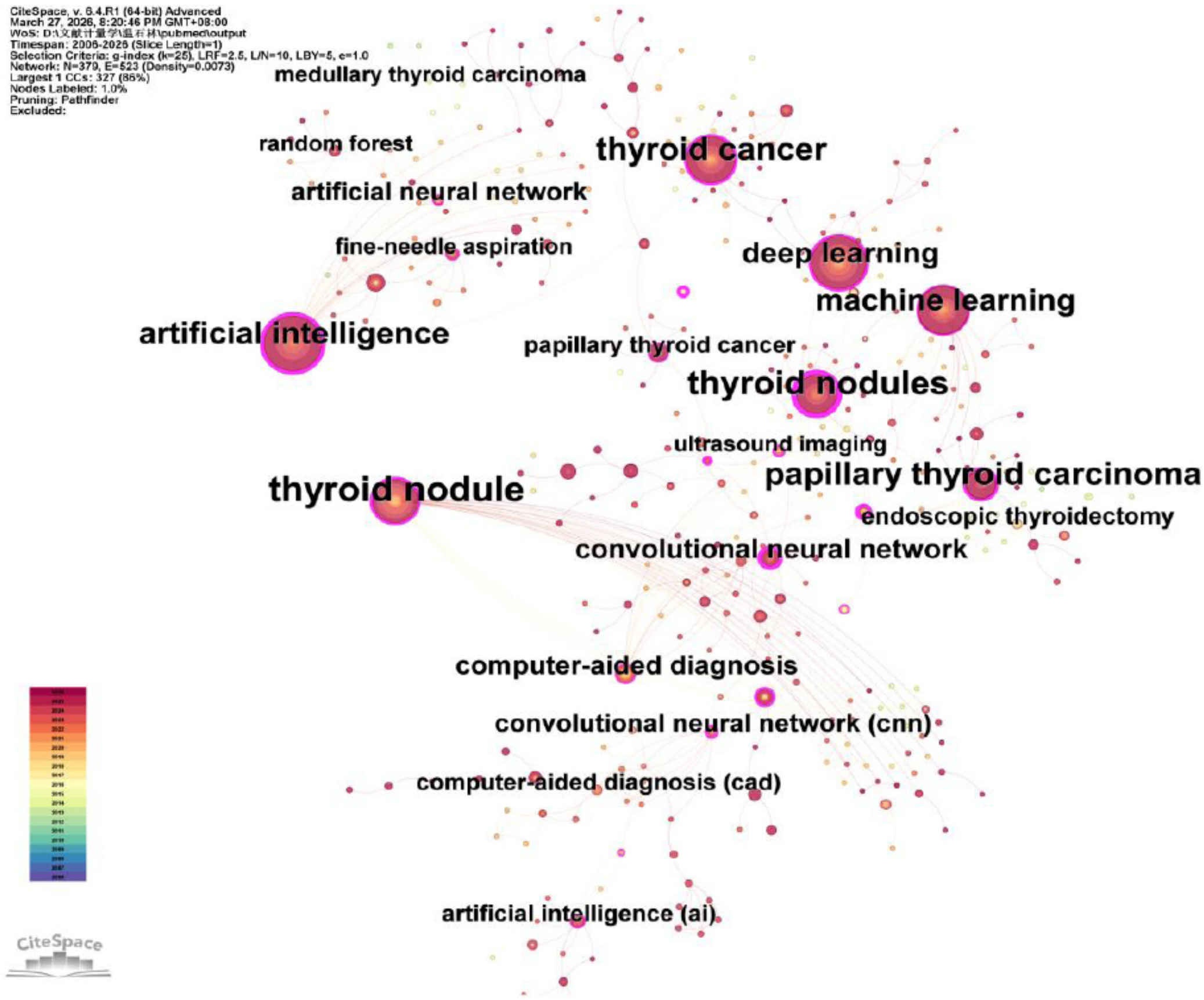

1. Co-occurrence of Pubmed keywords

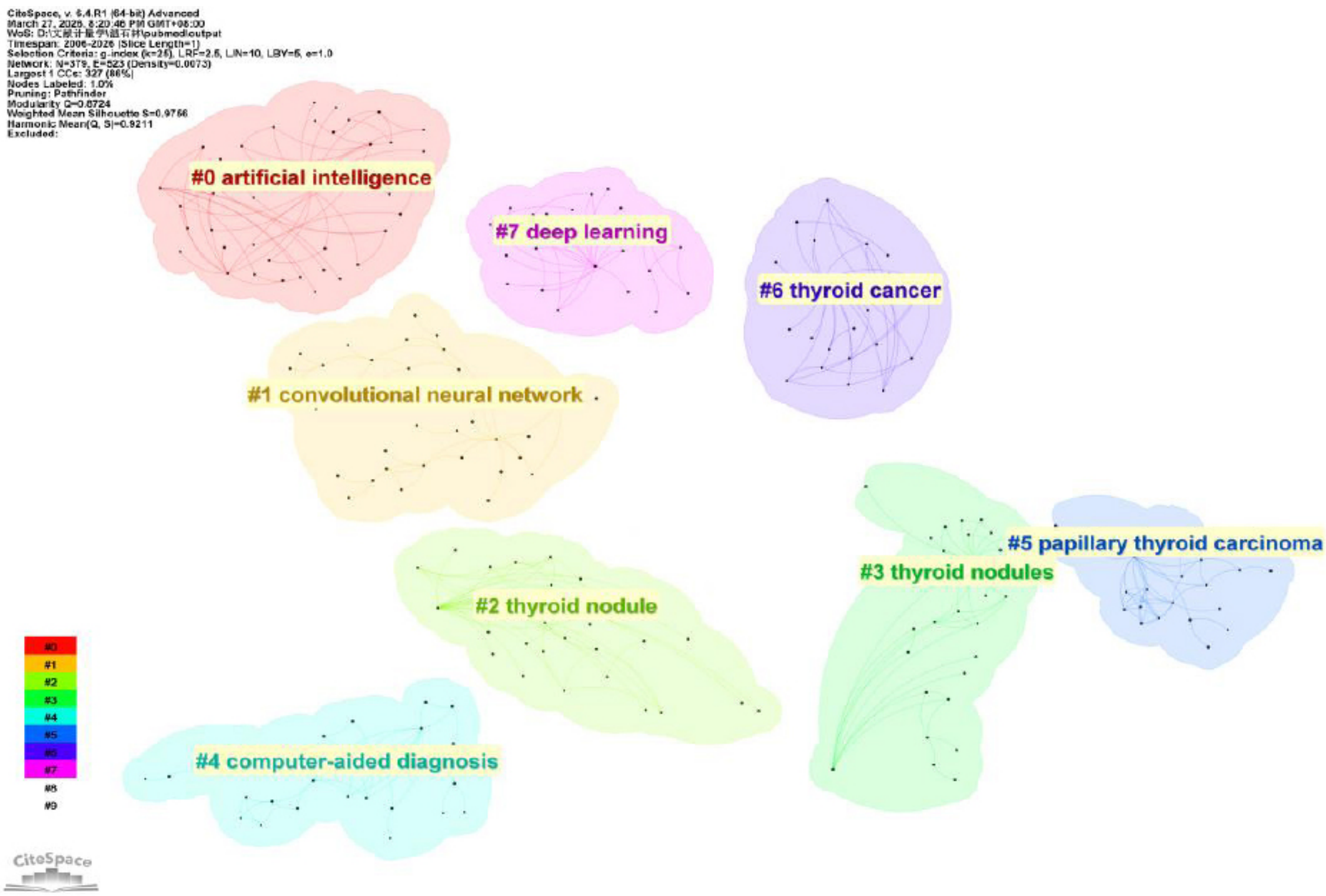

2.Pubmed Keyword clustering

2. The top ten keywords of the cluster

No.	Documents	Year	Keywords
1	150	2017	Artificial intelligence
2	132	2017	Deep learning
3	117	2016	Machine learning
4	107	2014	Thyroid cancer
5	100	2014	Thyroid nodule
6	82	2016	Thyroid nodules
7	50	2013	Papillary thyroid carcinoma
8	21	2016	Convolutional neural network
9	19	2016	Papillary thyroid cancer
10	17	2018	Lymph node metastasis

© 2026 The Author(s). Published by Bentham Science Publisher.

This is an open access article distributed under the terms of the Creative Commons Attribution 4.0 International Public License (CC-BY 4.0), a copy of which is available at: https://creativecommons.org/licenses/by/4.0/legalcode. This license permits unrestricted use, distribution, and reproduction in any medium, provided the original author and source are credited.

HOW TO CITE:
Wen S, Qian W, Su Y, Cao X, Chen T, Gong W, Lei X. Dual-Database Bibliometric Analysis Combined with Gephi-Based Network Visualization of Artificial Intelligence Applications in the Identification and Diagnosis of Thyroid Space-Occupying Lesions. Curr Med Imaging, 2026; 22: e15734056466850.
http://dx.doi.org/10.2174/0115734056466850260610101215
